# Understanding the Occupational Adaptation Process and Well-Being of Older Adults in Magallanes (Chile): A Qualitative Study

**DOI:** 10.3390/ijerph16193640

**Published:** 2019-09-27

**Authors:** Oskarina Palma-Candia, César Hueso Montoro, Celia Martí-García, Manuel Fernández-Alcántara, Concepción Petra Campos-Calderón, Rafael Montoya Juárez

**Affiliations:** 1 Faculty of Health Sciences. University of Magallanes, Punta Arenas 6200000, Chile; oskarina.palma@umag.cl; 2 Faculty of Health Sciences, University of Granada, 18016 Granada, Spain; rmontoya@ugr.es; 3 Faculty of Health Sciences, University of Málaga, 29071 Málaga, Spain; 4 Department of Health Psychology, Faculty of Health Sciences, University of Alicante, 03690 Alicante, Spain; mfernandeza@ua.es; 5 University of Granada, 18071 Granada, Spain; concha_campos@hotmail.com

**Keywords:** adaptation, psychological, aging, life change events

## Abstract

*Background*: Aging and longevity are important topics nowadays. *Purpose*: To describe how older adults perform the occupational adaptation process in the extreme region of Magallanes (Chile), and to identify the factors that might contribute to successful occupational adaptation and well-being. *Method*: Qualitative study, with a phenomenological interpretative approach. In-depth interviews were carried out with 16 older adults, with high or low levels of well-being, assessed with the Ryff Scale. An inductive content analysis according to Elo and Kyngäs was performed. *Findings*: Resilience, self-esteem and interdependence with significant others are key elements that promote well-being. Participants develop strategies to minimize the effects of environmental factors. The occupation’s function in terms of socialization, use of time, and social participation is revealed as a conditioning factor of occupational adaptation. *Implications*: Interventions with older people to achieve a successful occupational adaptation process must take into consideration the commitment to meaningful activities.

## 1. Introduction

The aging and longevity of the population, a current concern at both the regional and global level, has led international organizations to guide their policies to achieve greater well-being at an older stage of life by promoting healthy aging, within which they encourage significant participation in activities [1]. From the perspective of therapy and occupational science, human beings are occupational beings [2] and to the extent that they manage to engage in meaningful activities, this affects their health and well-being [3].

Old age represents a life stage, culturally devalued in some western countries, as it is associated with important biopsychosocial changes such as dependence, diseases, loss of loved ones, changes in social roles and retirement, among others. However, how to live this stage depends on multiple factors, among which personal and social factors become important, as well as the way of coping with life-changing situations [4,5]. Occupational science investigates the impact of involvement in meaningful activities on the health and well-being of older adults [3]. In turn, qualitative studies have linked participation in occupations to greater well-being, associated with maintaining capabilities, autonomy, and the meaning given to activities [6,7].

Occupational adaptation is the capacity to construct a positive occupational identity and achieve occupational competence [8]. It is not only developed as a reaction to adverse situations [9], but it is also an everyday strategy to advance in occupational options, reinforcing one’s self-concept and well-being [10]. A series of factors have been found to influence this process: (i) Personal, including biological, psychological, social, and health aspects; (ii) Environmental, including culture and geographical, climatic, and economic aspects, among others; (iii) Occupational, including aspects such as form, function, and meaning [11]; in addition to the characteristics of the activities and skills themselves [12]. These factors have also been described as the person–environment–occupation model (PEO) [13], which explains how the interaction among these factors influences adaptation processes [14].

Few studies have specifically focused on the manifestations of the adaptation process in older adults and the factors that might influence this process [10]. In addition, a recent scoping review [15] showed that most of the published research that focuses on occupational adaptation has been conducted in the United States, Australia, Canada or Sweden, so it might be interesting to know how this concept applies in practical contexts in other parts of the world.

Aging in Magallanes and the peculiarities associated with its extreme geographical and climatic situation is an issue that has not been studied in depth. It is important to highlight that Chile is the country with the greatest life expectancy in the region of Latin America [1] and the Chilean Magallanes and Antarctic region are two of the regions with the highest levels of aging in the country [16]. In terms of geography, it occupies an extreme position in the Latin American southern cone, which gives it a role as an entrance platform to the Antarctic continent and it is an extensive unpopulated territory, accessible mainly by land or sea. Its climate is characterized by its low temperatures, strong winds, and intermittent rain and snow throughout the year [17].

Although the impact of occupational therapy and participation in activities in similar climatic contexts in older people has been studied [18], as far as we know, this is one of the first studies conducted in the Chilean Magallanes and Antarctic Region that focuses on the process of occupational adaptation, and its relationship with well-being in the elderly population.

The aim of this study is to determine the factors that contribute to the occupational adaptation process in older adults in the extreme region of Magallanes (Chile) and identify the signs of success and well-being.

## 2. Materials and Methods

This is a qualitative phenomenological interpretative research. Phenomenology may be described as the study of concrete lived experiences [19]. There are two basic approaches to phenomenology: descriptive and interpretative. Interpretive phenomenology aims to understand and interpret participants’ experiences, rather than only describe these experiences [20].

### 2.1. Sample and Participants

Participants were chosen among a previous sample of 101 older adults between 64 and 75 years old from the Magallanes Region [21]. The selection criteria of the participants were the scores on well-being on the Spanish version of the Ryff Psychological Well-Being Scale (PWB) [22,23,24]. This scale measures six aspects of well-being and happiness: autonomy, environmental domain, personal growth, positive relations with others, purpose in life, and self-acceptance. The scale consists of 39 items, that could be rated according to a Likert scale from 1 (absolutely agree) to 6 (absolutely disagree) and its Spanish version showed good reliability (Cronbach α = 0.64 to 0.83, depending on dimensions). 

The eight elderly from the previous sample that presented with the highest scores on the PWB scale, and the ones that had the eight lowest scores were interviewed. As exclusion criteria, none of the older adults selected worked part-time at the time of the data collection. Other characteristics of the participants were sex, marital status, and living situation (whom they live with) (Table 1).

### 2.2. Data Collection

A semi-structured interview guide was developed, oriented from theoretical aspects of the science of occupation, to explore the factors that could contribute or hinder the process of occupational adaptation. The interview guide was initially tested with 4 older adults, who were not study participants. These interviews were held between May and June 2016 in the participants’ homes by an occupational therapist with previous training in qualitative research. Interviews lasted for an average of one hour. The study was approved by the Ethics Committee of the University of Magallanes and the University of Granada (code: 117/CEIH/2015), and all of the participants, after being informed about the nature and purpose of the study, signed an informed consent form. The final interview guide appears in Table 2.

### 2.3. Data Analysis

Each interview was recorded, transcribed, and integrated into the Atlas ti 7.0 © software (Atlas.ti Scientific Software Development GmbH, Berlin, Germany). The analysis followed the sequence described by Elo and Kyngäs [25]. First of all, the researchers read all the interviews in order to become familiar with the data. Significant meaning text units (quotations) were identified and assigned to create codes that described their content. The first researcher performed a preliminary coding, which was discussed, modified and agreed upon by the three researchers, based on the study objectives and fundamental concepts of occupational adaptation. Finally, these codes were grouped into themes and subthemes, which were related to each other to increase understanding of the phenomenon. Thematic saturation was achieved since no new themes were identified in the last interviews analyzed and the phenomenon was sufficiently explained. Thematic saturation focuses on the identification of new codes or themes, rather than existing theoretical categories [26]. 

## 3. Results

**Figure 1:**
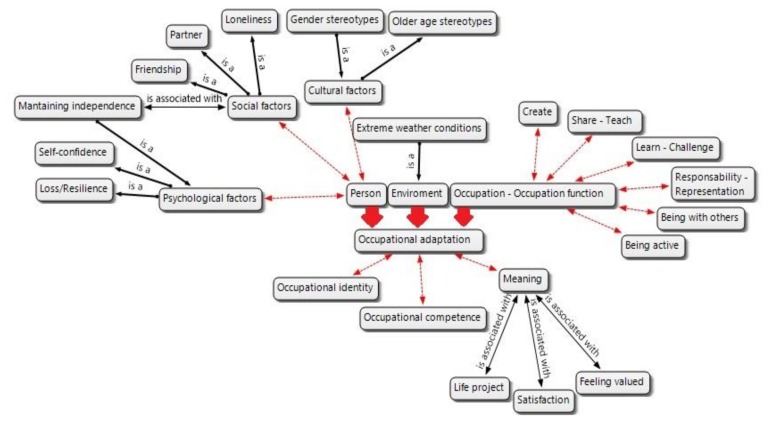
General map of codes and categories.

The occupational adaptation of elderly in the region of Magallanes is conditioned by three types of factors related to the person, the environment, and the occupation. The elderly who are occupationally adapted in this region show a series of manifestations that have been grouped into the following themes: occupational identity, feelings of occupational competence, and perception of meaning in their occupations. Figure 1 shows the general map of themes, sub-themes, and codes. A numeric code has been used to identify quotations: the first number in brackets after quotations indicates the participant, and the second number indicates the quotation number of each interview.

### 3.1. Factors Related to the Person

#### 3.1.1. Psychological Factors

The majority of the interviewees refer to having experienced adverse situations and losses, highlighting the capacity for resilience and the importance of the family as support: “*When I was 14, I could not continue studying, because my father died in an accident. It was so traumatic, that, until today, I do not like Christmas, and if I celebrate it, I celebrate it just for my children and my husband (…), well, but over time that could be overcome*” (6:52).

One of the most significant losses referred to by the participants occurs when their children grow up and leave the region to find a job or study in other cities sometimes 1000 or 3000 km from Magallanes: “*It hurts a lot … I cried a lot, but then I said no. It can’t be… they also have the right to live their lives too… so it’s sad not to be able to have them around*” (12:23).

Participation in meaningful occupations helps them to feel self-efficacious and valued. Achieving skills and mastery in the development of an occupation promotes a feeling of self-confidence and security, and it stimulates the continuation and enjoyment of the activity: *“We were talking about the tango… That is what gives me satisfaction. Because if you feel embarrassed, you will never do anything right. You will hide, and feel that you are going to do it wrong. You have to believe in yourself, in your abilities”* (3:12).

Maintaining independence is a recurring theme in the speeches of the interviewees, visualizing it as the possibility of continuing to participate actively and autonomously in society. This stresses the relevance of emotional interdependence, that is, the importance assigned to significant others when it comes to continuing or stopping activities that are important to them: *“My son doesn’t like me to go out alone at all. When I go out, he gets angry. Then I hardly do anything outside the house”* (9:28).

#### 3.1.2. Social Factors

Friendship is one of the most recognized relationships by the interviewees, generating help, support, company and allowing the development of co-occupations. Sometimes when the significant other is no longer there, people stop carrying out their activities and these seem to lose meaning: “ *I started going to the club with a friend who invited me, with her we participated in activities and went to other places as well. But since she died years ago, I’ve never gone back to the club or been involved in those things”* (9:27).

Having a partner seems to be a fundamental element for some participants. Without a partner, some participants find it difficult to organize their routine or find a reason to plan new activities. Some of the interviewees, specifically widowers, are still waiting to find a new partner or are happy to have found one, describing the importance of being accompanied at this stage: *“I would like to have a partner, but it is not as easy as saying “I want a partner” and that’s it… It is life that gives it to you or not”* (15:33). 

Loneliness is also important in the speeches of the interviewees, and sometimes also seems to be associated with feelings about the loss of loved ones or as a result of personal decisions. The occupations in which they get involved can enhance or lessen this feeling of loneliness: *“In my case I never got married I did not have children…and I have learned to enjoy what life gives me. Now that I retired I discovered this new activity, which I like. I love”* (8:45).

#### 3.1.3. Cultural Factors

During the interviews, gender biases were observed, some of which relegate women to a less appreciated sphere within society, linking household chores as something with lesser value, which is observed in phrases such as: “*No. I’m just at home. I don’t work or do anything*” (13:5). There were also stereotypes about old age, which allude to themes such as abandonment, a feeling of being a nuisance to others, or that it is not worth making major changes, or that things are unalterable: “*I spend a lot of time alone. They [sons] are in their things do not care about me, and I wonder why I should go … My son works, his wife works, they are always busy. If I go, I will be a nuisance so I do not visit them. And every day that passes, I find it more difficult to go*” (15:8).

### 3.2. Factors Related to the Environment

For some interviewees, the extreme weather conditions in the region represent a barrier to participating in outdoor activities or leaving home. Some point out that their attitude toward climate has changed over the years, due to their physical condition or the loss of certain skills associated with aging: “*When you start to age, you start to lose agility, then you can’t run on the frost, you can’t do certain things… when I was young… the frost didn’t stop me, nor the bad weather. I came out the same, but now with age all the pains start*” (16:42).

However, for others, extreme temperatures are accepted as a characteristic of the region to which they are already accustomed and have adapted: *“No, the weather doesn’t limit me because I go out with thunder or lightning, I’m not afraid of the cold, and if I feel cold I put on thicker clothes, but I still go out”* (12:29). The available infrastructure does not always take into account the needs of people with reduced mobility, as there are few roofed spaces where participants can perform some of their occupations of interest during winter: *“Suppose there is a tremendous snowstorm or frost (…) I can try to reach the place, but if it does not have railing I cannot hold or move on my own”* (14:29).

### 3.3. Factors Related to the Occupation

The participants’ accounts reveal the function of occupation, mainly associated with aspects such as socialization, the use of time and social participation. It is difficult to find a common pattern among occupations and why some occupations turn out to be more significant than others. However, it is important to note that those who were active and participated in occupations of more personal meaning coincided with those who had higher levels of well-being according to the Ryff scale, while those who did not develop activities outside the home or only attributed minimal meaning to their participation in occupations, in terms of the importance of using free time or staying physically and mentally active, presented the lowest levels in the same scale.

Some participants have found their significant occupations through artistic expression, it being significant for them to be able to share their creations with the community: *“When we dance the cueca or tango, we always go out into the community and share our art with other people and that is very gratifying”* (3:39).

Some participants begin to learn different occupations, motivated by the challenge. The interviews reveal that, for some, learning new occupations has allowed them to maintain their development and growth: *“I am always trying to learn only for the satisfaction of learning. It is an attitude. You start to discover that you can do something and then you want to learn to do more things”* (8:37).

For others, the meaning they refer to is related to giving or teaching something to others, which is associated with having a social role valued by society: *“As a teacher you work with the best in society, young people who are learning, and it is important to feel that you help them in this”* (4:24).

Some have been or are part of the board of community organizations. These experiences are significant occupations and spaces in which they validate the responsibility of having representation roles in their community: *“Now they have nominated me as a candidate to represent the neighbors in civil society. Those who are elected can meet with the mayor and present the concerns of their group”* (7:36).

In some cases, more than the occupation itself, the importance of being with others, sharing and socializing, regardless of the activities that can be carried out together, becomes more valuable: *“I really like being with friends, with the community group, since at home or with the family, children, grandchildren are no longer very interested in what one does”* (2:32).

Finally, the participants’ accounts reveal that performing their occupations is related to the desire to be active, both physically and mentally: *“Driving a taxi is something new; you have to be alert all the time, have good reflexes for driving… It has to do with cognitive tasks”* (8:29).

#### Signs of Occupational Adaptation

The interviewees related their current occupations with the activities carried out in their childhood with their parents, emphasizing that these activities were always part of them, but now they are able to recognize them as part of their identity and with their abilities, and that now they practice these with special meaning: *“When I was a child, at home, my father and mother sang, they did artistic things, such as dancing and singing frequently. I assimilated all this without knowing it, and it is now in my old age that I realize that these activities are part of me”* (1:31).

Some older people maintain their occupational identity, carrying out occupations similar to those they did while they were still working: *“The profession I chose as a pharmaceutical chemist is similar to other activities with which I have now reconnected, for example, cooking, or painting with oil I am doing things related to what I was doing before, I like that”* (1:11). However, others are able to discover new occupational options, carrying out different activities that allow them to discover other aspects of their identity: *“This new activity [driving a taxi], changed my life, now I move to different places, knowing different people, after being in education for 42 years, now I realize that I was locked in a school for years”* (8:28).

Occupational competence can be seen in their perception of continued personal growth and development, the use of their capabilities and potential, and the development of a positive sense of self-efficacy: *“I have been a teacher for 51 years, and I am preparing classes for them as if I were just out of the University; I mean, I don’t get exhausted. Why don’t I get exhausted? Because the effort I make is minimal because I like what I do”* (4:26).

Occupationally-adapted older adults consider that their participation in occupations is meaningful and makes sense in their life project. Some associate it with satisfaction obtained from their achievements, whereas others state that they feel happy and passionate about performing occupations they love: *“I don’t think there’s anything pending; I have done so many things and one has to feel satisfied; well, I can still manage to do a lot of other things”* (5:59).

Some interviewees also say they feel distinguished and valued in certain roles they have accepted in their community, referring to the satisfaction they derive from helping others: *“I feel satisfied when I do things for the neighbors, because it’s not for me, but rather for the neighbors. Helping others gives me satisfaction”* (7:37).

## 4. Discussion

This study aimed to determine the factors involved in occupational adaptation in older adults in Magallanes (Chile) and to identify the signs of success of this process. Participants who achieve a successful occupational adaptation process, construct a new occupational identity, that associated with a sense of efficacy and occupational competence, and who are committed to an occupation that is meaningful to them, are the ones that present higher indexes of well-being. 

Our results suggest, in line with previous studies [27], that self-esteem is one of the relevant factors related to the person, which is encouraged through participation in occupations from an identity construction perspective. Older adults in the present study increase their self-confidence and security in the performance of the occupation, promoting a sense of occupational efficacy, through the achievement of greater dominion in developing this specific occupation. This is relevant also for children [28].

Older adults from Magallanes reported that, as in other studies in similar populations, interdependence with significant others in performing co-occupations becomes an important activity [29]. Friendship is one of the most recognized relationships by the interviewees. As Coll-Planas et al. [30] also highlighted, friendship seems to be stronger and deeper than in other phases of life. This characteristic is associated with the interdependence that occurs in the development of co-occupations, as Pierce [31] points out, so that when a significant “other” is not there, the person might not continue to do these activities, or if he/she does, then the meaning may change. This finding is of great interest because social relations tend to decline in this stage of life [32]. 

Regarding the environmental factors, the participants perceived the geographical and climate characteristics, both as a facilitator or as a barrier to participation. The results of our study show that the characteristics of the region of Magallanes encourage the interviewees to develop adaptation strategies and strengthen aspects related to regional identity, reported in a previous study [33].

As cultural elements, stereotypes about old age appear repeatedly in the discourse of the interviewees, as they consider old age to be linked to illness that alters the life balance [34]. Chile also maintains inequalities based on gender. In their discourse, older people indicate that women must be limited to certain activities [35].

Considering the geographical and cultural characteristics of the region [33], its isolation and remoteness from the country’s major urban centers, usually as children grow up, they leave Magallanes in search of job opportunities or to pursue their studies, essentially constituting permanent emigration, which is a factor that has great impact on the elderly.

Various functions of occupation are associated with occupational adaptation processes. In our findings, some older adults learned different occupations, motivated by the challenge involved in setting new goals. As Jackson [36] and Stanley [37] point out, older adults conserve the motivation to accept risks. 

For other participants, the function of the occupation is linked to more specific needs, such as teaching or helping others and leading or directing organizations, as Zapata [38] also highlights. Likewise, older adults of Magallanes showed other factors of the occupation that the literature reported that might help to occupational adaptation, as a sense of social belonging [39], being valued by their peers, and personal efficacy in developing their occupations [8]. All these factors might influence their perception of well-being [40]. 

Based on the interaction among the diverse factors mentioned above, occupational adaptation processes related to well-being occur. As the results show, these processes are not only a reaction to adverse situations or illness, but they are also part of life development through adjustments in daily life.

Our findings show two manifestations linked to successful occupational adaptation in older adults in Magallanes: (1) the construction of a new occupational identity and (2) the perception of occupational competence, obtained through the evaluation of their self-efficacy in performing activities. Both concepts have been studied before in association with occupational adaptation processes [8,10,41,42]. As a new manifestation of this adaptation process, the present study proposed the meaningfulness of the occupation, which Larson et al. [43] refer to as subjective experience and personal value of participating in occupations in the context of real-life and culture. 

Our results suggest that the possibility of helping others play a key role in an occupation’s meaning, as Albolfathi et al. [44] points out. The occupationally adapted interviewees describe the sense and logic of participating in occupations and the value of integrating the occupations developed into their life project and sense of the future. 

### Study Limitations

Among the limitations of this study, it should be remarked that three older adults with low scores on well-being refused to participate, due to lack of interest or lack of time. In addition, it was not possible to obtain two groups of similar sociodemographic characteristics because the interviewees who obtained higher scores on well-being had higher educational levels. This point has been discussed in a previous study [21]. Other factors, such as age, should be considered for participant selection, due to the importance for occupational adaptation.

On the other hand, although different researchers have agreed on codes, themes, and sub-themes, these are conditioned by the semi-structured interview guide, so some relevant aspects of the occupational adaptation might not be included in the final version of the manuscript.

## 5. Conclusions

Older adults who achieve a successful occupational adaptation process, construct a new occupational identity, associated with a sense of efficacy and occupational competence, and are committed to an occupation that is meaningful to them, present higher indexes of well-being. Personal factors such as resilience, self-esteem, and relationships of interdependence with significant others are elements that favor well-being.

## Figures and Tables

**Table 1:** Participant Data.

Participant	Age	Sex	Occupation	Marital Status	Educational Level	Who They Live With	Job Situation	Perception Well-Being Ryff	Score Well-Being Ryff
1	75	M	Retired	Widower	University studies	Alone	Part time	High	214
2	73	F	Housework	Married	Basic studies	Spouse and family	No job	High	205
3	75	F	Retired	Married	University studies	Spouse	No job	High	200
4	72	M	Retired	Married	University studies	Spouse	Part time	High	202
5	70	F	Housework	Widow	Basic studies	Alone	No job	High	212
6	64	F	Housework	Married	Basic studies	Spouse and family	No job	High	204
7	70	M	Retired	Married	Basic studies	Spouse	No job	High	207
8	65	F	Retired	Single	University studies	Alone	Part time	High	204
9	77	F	Housework	Widow	Medium/Technic studies	Family	No job	Low	143
10	68	F	Housework	Married	Basic studies	Spouse	No job	Low	149
11	75	M	Retired	Married	No studies	Spouse	No job	Low	153
12	68	F	Housework	Married	Basic studies	Spouse and family	No job	Low	159
13	65	F	Housework	Married	Basic studies	Spouse and family	No job	Low	156
14	73	F	Housework	Widow	Basic studies	Alone	No job	Low	148
15	77	M	Retired	Widower	Basic studies	Alone	No job	Low	139
16	72	M	Retired	Married	No studies	Spouse and family	No job	Low	183

The scores on the Ryff, scale can vary from a minimum score of 39 to a maximum score of 234. We consider high perception to be above 200 points, and scores lower than 200 points to be a low perception, based on the average of the total sample of 101 participants.

**Table 2:** Semi-Structured Interview Guide.

1. Give me a short summary of the most important events in your life.
2. What do you currently dedicate your time to, in general, and what activities do you like to do?
3. In relation to the activities you do and those you no longer do, what factors do you think have influenced these choices?
4. From the activities you participate in, which ones would you say are the most important to you, and why?
5. How are these activities related to your well-being?
6. In general, what helps you, or makes it difficult to participate in the activities you want to do?

## References

[1] World Health Organization (2015). World Report on Ageing and Health.

[2] Yerxa E.J. (1998). Health and the human spirit for occupation. Am. J. Occup. Ther..

[3] Eakman A.M., Carlson M.E., Clark F.A. (2010). The meaningful activity participation assessment: A measure of engagement in personally valued activities. Int. J. Aging Hum. Dev..

[4] Cosco T.D., Wister A., Brayne C., Howse K. (2018). Psychosocial aspects of successful ageing and resilience: Critique, integration and implications. Stud. Psychol..

[5] Fontes A.P., Neri A.L. (2015). Resilience in aging: Literature review. Cienc. Saude Colet..

[6] Chilvers R., Corr S., Singlehurst H. (2010). Investigation into the occupational lives of healthy older people through their use of time. Aust. Occup. Ther. J..

[7] Liddle J., Gustafsson L., Bartlett H., McKenna K. (2012). Time use, role participation and life satisfaction of older people: Impact of driving status. Aust. Occup. Ther. J..

[8] Kielhofner G. (2008). A Model of Human Occupation: Theory and Application.

[9] Williams S., Murray C. (2013). The lived experience of older adults’ occupational adaptation following a stroke. Aust. Occup. Ther. J..

[10] Nayar S., Stanley M. (2015). Occupational adaptation as a social process in everyday life. J. Occup. Sci..

[11] Clark F. (2006). One person’s thoughts on the future of occupational science. J. Occup. Sci..

[12] Bagatell N., Womack J.L. (2016). Human capacity for action as core content in occupational science education. J. Occup. Sci..

[13] Law M., Cooper B., Strong S., Stewart D., Rigby P., Letts L. (1996). The person-environment-occupation model: A transactive approach to occupational performance. Can. J. Occup. Ther..

[14] Broome K., McKenna K., Fleming J., Worrall L. (2009). Bus use and older people: A literature review applying the person-environment-occupation model in macro practice. Scand. J. Occup. Ther..

[15] Johansson A., Fristedt S., Boström M., Björklund A. (2018). The use of occupational adaptation in research: A scoping review. Occup. Ther. Health Care.

[16] Ministerio de Desarrollo Social (2015). Encuesta CASEN 2013: Adultos Mayores, Síntesis de Resultados. http://observatorio.ministeriodesarrollosocial.gob.cl/documentos/Casen2013_Adultos_mayores_13mar15_publicacion.pdf.

[17] Arenas Vásquez F., Aliaga Bustos G., Marchant Santiago C., Sánchez Acuña R. (2005). El Espacio Geográfico Magallánico: Antecedentes Acerca de su Estructura y Funcionamiento. Tiempo Espac..

[18] Johansson A., Björklund A. (2015). The impact of occupational therapy and lifestyle interventions on older persons’ health, well-being, and occupational adaptation. Scand. J. Occup. Ther..

[19] Van Manen M., Adams C.A., Peterson P., Baker E., McGaw B. (2010). Phenomenology. International Encyclopedia of Education.

[20] Matua G.A., Van Der Wal D.M. (2015). Differentiating between descriptive and interpretive phenomenological research approaches. Nurse Res..

[21] Palma Candia O., Hueso Montoro C., Ortega-Valdivieso A., Montoya Juárez R., Cruz-Quintana F. (2016). Wellbeing of Chilean older adults is associated with group participation. Rev. Med. Chil..

[22] Díaz D., Rodríguez-Carvajal R., Blanco A., Moreno-Jiménez B., Gallardo I., Valle C., Van Dierendonck D. (2006). Adaptación española de las escalas de bienestar psicológico de Ryff. Psicothema.

[23] Van Dierendonck D. (2004). The construct validity of Ryff’s scale of psychological well-being and its extension with spiritual well-being. Pers. Individ. Dif..

[24] Ryff C.D. (1989). Happiness is everything, or is it? Explorations on the meaning of psychological well-being. J. Personal. Soc. Psychol..

[25] Elo S., Kyngäs H. (2008). The qualitative content analysis process. J. Adv. Nurs..

[26] Saunders B., Sim J., Kingstone T., Baker S., Waterfield J., Bartlam B., Burroughs H., Jinks C. (2018). Saturation in qualitative research: Exploring its conceptualization and operationalization. Qual. Quant..

[27] Howie L., Coulter M., Feldman S. (2004). Crafting the self: Older persons’ narratives of occupational identity. Am. J. Occup. Ther..

[28] Viana Moldes I., Pellegrini Spangenberg M., Viana Moldes I., Castellanos Ortega M.C., Polonio López B. (2008). Consideraciones contexturales en la infancia introducción al desarrollo del niño. Terapia Ocupacional en la Infancia.

[29] Van Nes F., Jonsson H., Hirschler S., Abma T., Deeg D. (2012). Meanings created in Co-occupation: Construction of a late-life couple’s photo story. J. Occup. Sci..

[30] Coll-Planas L., Del Valle Gomez G., Bonilla P., Masat T., Puig T., Monteserin R. (2017). Promoting social capital to alleviate loneliness and improve health among older people in Spain. Health Soc. Care Commun..

[31] Pierce D. (2009). Co-Occupation: The challenges of defining concepts original to occupational science. J. Occup. Sci..

[32] Tomioka K., Kurumatani N., Hosoi H. (2016). Social participation and the prevention of decline in effectance among community-dwelling elderly: A population-based cohort study. PLoS ONE.

[33] Molina C.W. (2011). Identidad regional en Magallanes, sus expresiones simbólicas y territoriales. Magallania.

[34] De Haro Honrubia A. (2014). El estigma en la vejez. Una etnografía en residencias para mayores. Intersecc. Antropol..

[35] Vega Carvajal D.I. (2009). Los Discursos Sobre la Calidad de Vida de Hombres y Mujeres Mayores, Desde una Perspectiva de Género. Master’s Thesis.

[36] Jackson J., Zemke R., Clark F. (1996). Living a meaningful existence in old age. Occupational Science: The Evolving Discipline.

[37] Stanley M. (2006). Older People’s Understandings and Perceptions of Well-Being: A Grounded Theory. Ph.D. Thesis.

[38] Zapata Farías H. (2001). Adulto mayor: Participación e identidad. Rev. Psicol..

[39] Hammell K.R. (2014). Belonging, occupation, and human well-being: An exploration. Can. J. Occup. Ther..

[40] Gallagher M., Muldoon O.T., Pettigrew J. (2015). An integrative review of social and occupational factors influencing health and wellbeing. Front. Psychol..

[41] Christiansen C. (2000). Identity, personal projects and happiness: Self construction in everyday action. J. Occup. Sci..

[42] Nelson D.L., Jepson-Thomas J. (2003). Occupational form, occupation. Perspectives in Human Occupation: Participation in Life.

[43] Larson E., Wood W., Clark F., Blesedell Crepeau E.B., Cohn E.S., Boyt Schell A. (2005). Ciencia ocupacional: Desarrollo de la ciencia y la práctica de la ocupación a través de una disciplina académica. Willard & Spackman Terapia Ocupacional.

[44] Abolfathi Momtaz Y., Ibrahim R., Hamid T.A. (2014). The impact of giving support to others on older adults’ perceived health status. Psychogeriatrics.

